# Chiefs and floods: hybrid governance and co-production of flood risk adaptation in Tamale, Ghana^1^

**DOI:** 10.1080/1523908X.2024.2410899

**Published:** 2024-10-10

**Authors:** Samuel Agyei-Mensah, George Owusu, Cynthia Awuni, Ben Howard, Issahaka Fuseini, Wouter Buytaert, Frans Berkhout

**Affiliations:** a Department of Geography and Resource Development, University of Ghana, Legon, Ghana; b Centre for Migration Studies, University of Ghana, Legon, Ghana; c Department of Civil and Environmental Engineering, Imperial College London, London, UK; d Department of Adult Education and Human Resource Studies, University of Ghana, Legon, Ghana; e Department of Geography, King’s College London, London, UK

## Abstract

Climate change is changing physical and social risks facing people in African cities. Emerging awareness is beginning to stimulate a wide range of adaptive responses. These responses are playing out in a complex institutional and governance context which shape their effectiveness and legitimacy. Employing a hybrid governance approach, we investigate the development of flooding and flood protection in the context of urban development in Tamale, Ghana. We argue that the interplay between traditional and state-based authority shapes the market for land, the regulation of land use and the provision of urban services, including flood protection. Hybrid governance influences the types of knowledge applied to urban problem-solving, the legitimacy of choices made, the human and other resources that can be deployed in building community resilience and the willingness to act in the provision of public goods by communities. We suggest how the existing hybrid governance setting could be strengthened to achieve more effective and legitimate adaptation to dynamic flood risks under climate change in Tamale, with lessons for other West African contexts.

## 1 Introduction

Climate change is transforming hazardscapes faced by people in African cities, playing into social and institutional contexts that shape vulnerabilities, as well as capacities to adapt. In urban systems, the ownership of land and land-use planning play a fundamental role in shaping climate-related risks, including flooding. How land is developed can affect the generation and propagation of flood waters, especially for flash flooding. The way that land is allocated leaves some people more exposed than others.

In West African settings, land ownership and planning are typically negotiated within complex ‘hybrid’ institutional contexts, including state and traditional authorities, civil society organisations, together with the occupiers of land, including farmers, businesses, and householders. These are dynamic settings, with processes of urbanisation unfolding in distinct historical and cultural contexts, coming to be influenced by emerging climate-related risks. In this paper, we aim to contribute to understanding how flood risk is perceived and framed, how this has led to action to build flood resilience in communities, and to suggest how new approaches co-produced by empowered hybrid governance settings could reinforce resilience under climate change. We take the northern Ghanaian city of Tamale, a dynamic urban centre facing endemic and increasing flood risk, as a case through which to analyse indigenous knowledge, legitimacy, resource mobilisation and action in response to climate change-influenced flood risks.

The impact of extreme weather events and flood risks affecting urban systems in sub-Saharan Africa has attracted more sustained attention in recent years (Serdeczny et al., 2017; The World Bank, 2022). These assessments have sought to quantify risks and potential adaptation responses and policies. To date, there has been limited attention paid to the institutional and governance settings in which climate-related risks emerge and which influence social and technical adaptation responses.

To address this gap, there is an opportunity to draw on scholarly literatures which have theorised and analysed governance in post-colonial West African contexts. We draw on a tradition of research into *hybrid governance regimes* in the provision of public goods, such as flood protection (Sklar, 2003). Local and regional governance does not conform to classical Weberian ideas about public good provision (for instance, protection from natural harms like flooding). Instead, other forms of authority share roles in the provision of public services, including customary or traditional authorities (community elders, headmen, clan chiefs, healers, religious leaders) and social structures (extended families, clans, tribes, religious brotherhoods). We argue that the complex interplay between state and traditional authorities can explain the pattern of natural hazard risks faced by communities, its understanding in local contexts, as well as the capacity and willingness of social actors (households, businesses, communities, cities) to respond to emerging climate risks. Our contribution to the hybrid governance literature is to go beyond its use to diagnose social and political orders and processes, and towards employing such insights to develop normative proposals for the implementation of flood protection measures in Tamale. We suggest how effective and legitimate climate and flood risk management could be more effectively co-produced by empowering traditional authorities in shaping and mediating community responses.

Traditional authorities and structures bring four ‘assets’ to local governance and action. First, they contribute local, indigenous and customary knowledge to the perception, framing and analysis of flood risk and options for managing risks (Hosen et al., 2020; Makondo & Thomas, 2018; Mekonnen et al., 2021; Tugjamba et al., 2023). In a local context, understanding of flood risks is likely to be shaped by customary knowledge as well as knowledge claims from technical, scientific sources. Second, the legitimacy of collective or private action in response to flood risks will be influenced by the opinions and practices of traditional rulers and leaders responding to local communities. Without confirmation about the significance of the problem and appropriateness of action by traditional rulers and leaders, state action on the ground will be difficult. Third, local communities have material, organisational and cultural resources that contribute to responses to flood risks, including customary ownership of land. Deployment of resources will be contingent on the degree to which traditional authorities can coordinate and motivate communities. Ray has argued that adding the legitimacy resources of traditional authority to those of the state facilitate compliance, co-operation, and mobilisation of communities that are both citizens of the state and subjects of traditional leaders (Ray, 1996). Finally, there is the ‘willingness to act’. Even if there is shared understanding of risks, without agreement about the value of private or collective action, little is likely to happen. Traditional authorities can play a role in aligning local interests and dispute resolution to enable collective action and distribute benefits.

In this paper, we analyse the relationship between planning authorities – principally Tamale Metropolitan Assembly (TaMA) – and traditional authorities and local communities as they shape flood risks and flood risk management in Tamale. Land governance is central to flood management, since flood risk and flood protection measures are spatially defined and because the capacity to change land-use depends on traditional authorities. Land is customarily owned, allocated and regulated by a network of local chieftaincies in northern Ghana (Boateng & Larbi, 2021; Tieleman & Uitermark, 2019). Planning authorities, who have the task of implementing national policies, do so in collaboration with traditional authorities, assembly members (local government community representatives) and civil society. In practice, much land allocation proceeds in consultation with traditional authorities, bypassing formal public planning authorities and leading to urban development that does not reflect planning and flood risk management policies. While metropolitan and district authorities have formal political responsibility for providing flood protection and resilience to communities, we observe a hybrid provision of this service, with traditional authorities and communities playing significant and distinct roles.

The paper has seven sections. In the following section, we summarise briefly the literature on hybrid governance regimes. Section 3 summarises the historical development of Tamale, including the evolution and interplay between state and traditional authorities, since the end of the nineteenth century. Section 4 sets out our methods. Section 5 provides an analyses of the role of traditional authorities in local and community responses to flooding and flood risk. Section 6 assesses climate adaptation strategies drawing on the opportunities offered by combining traditional and state authority. We conclude by arguing for a stronger role for local communities in co-producing appropriate responses to climate-influenced flood risk, with empowered and informed traditional authorities playing a significant intermediary role.

## 2 Hybrid governance regimes in Ghana

British colonial administrations governed Africa by co-opting traditional authorities to administer the affairs of local communities (Acemoglu et al., 2014; Gocking, 1994; Mawuko-Yevugah & Attipoe, 2021). Under this arrangement, traditional authorities performed tasks ranging from mobilisation of labour for public works, land administration, revenue collection through taxation, and maintaining law and order in areas under their jurisdiction. Post-colonial governance in West Africa sought at the outset to distance itself from traditional authority, with early nation building shaped by objectives of creating modern, centralised, unitary states. In practice, Weberian assumptions about public service provision, including flood risk management, as being offered primarily through a well-resourced and legitimate state do not hold in many African contexts (Boege et al., 2008; Dahlberg & Söderberg, 2023; Post et al., 2017; Ubink, 2008).

Instead, public goods and services are provided by configurations of state and non-state actors, sometimes including clandestine actors. There are broadly three reasons: the emergence of relatively weak or fragile public administrations in the post-colonial period which were poorly-resourced and did not command universal legitimacy; the emergence of alternative authorities and actors (including traditional authorities, civil society organisations and private sector) as alternative sources of services provision into the space left by weak states; and the development of market liberalisation policies from the 1970s onwards, often under pressure of international lenders, donors and advisors, including international NGOs, which further weakened state capacities and eroded legitimacy.

These developments have unfolded in distinctive ways across national and local contexts, but in many parts of Africa they have created multilateral or *hybrid* governance regimes in which traditional authorities, civil society and private actors share in the co-production of public services with the state (Abrefa Busia & Osei-Wusu Adjei, 2020; Ostrom, 1996). Ostrom argued that, especially at the local level, there are significant opportunities for complementarities through a stable, polycentric organisation of public goods provision (including flood resilience and disaster response), with state and non-state actors playing distinct roles. Later research has found that these contexts are typically complex and fluid, involving substantial negotiation and ambiguity for social actors concerned (Söderbaum, 2004; Ubink, 2009). They involve the interplay and entanglement of contrasting rationalities or logics of governance. For instance, while public administrations may work to a logic of technocratic allocation of resources commanding democratic consent, traditional authorities may work to customary norms, knowledges, and familial and elite networks. More critical assessments have argued that hybrid regimes are themselves frequently in tension, that legitimacy may be contested and that they can lead to new forms of conflict, exclusion and inequality (Akaateba et al., 2021; Barry & Danso, 2014; Kirst, 2020; Meagher, 2012; Meagher et al., 2014; Siiba et al., 2018; Yeboah & Shaw, 2013). While hybridity describes the structure and process of governance, it also implies a new series of problems and challenges to the governance and provision of public goods.

## 3 Evolution of hybrid public services provision in Tamale

**Figure 1:**
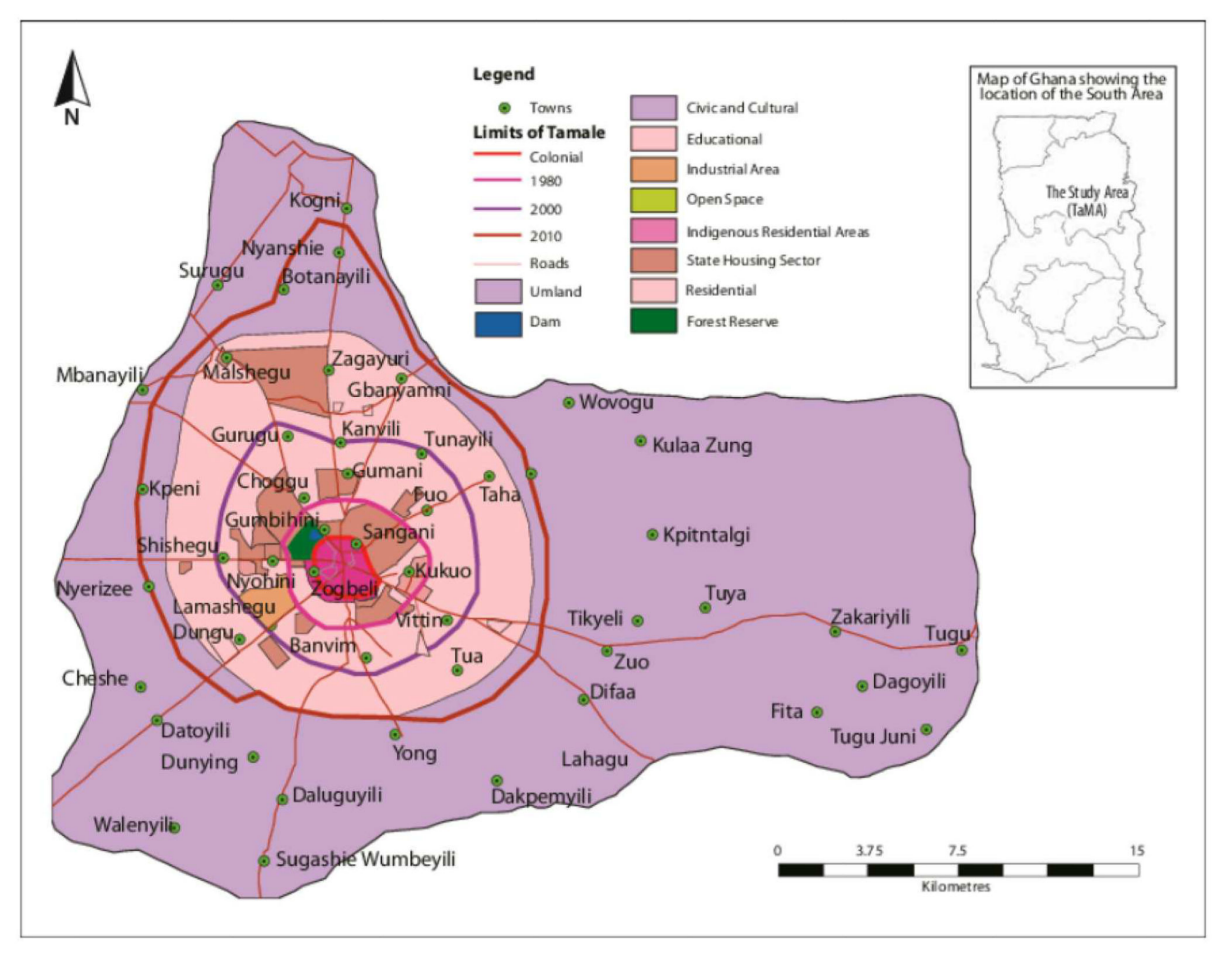
Map of Tamale (in supplemental material).

Tamale is the capital city of the Northern Region of Ghana (See Figure 1).^1^ It is one of the fastest growing cities in Ghana with a total population of 720,000 in 2021 (Ghana Statistical Service, 2021), having tripled in size in the past 25 years. More than 80% of residents of Tamale are Dagomba people and 90% are Muslims. Tamale experiences a tropical wet and dry climate with seasonal variations in temperature (Amankwaa and Gough 2023). Flooding (*Kokpegu*) is a major seasonal hazard in the city, causing disruptions in livelihoods and health, and occasional fatalities (Gough et al., 2019).

The city of Tamale was created under British colonial rule, following relocation of the administrative capital for the Northern Territories of Ghana from Gambaga in 1907. The new city’s founding aimed to establish a colonial administrative and mobilisation centre accessible to the wider region, offering faster communication between the southern and northern Ghana. Tamale offered a locational advantage and had better water resources than Savelugu, another candidate. Tamale grew steadily in the pre- and post-colonial periods. Today, Tamale is a cosmopolitan city, and its growth presents challenges for urban governance in terms of the provision of urban infrastructure and services, including water, housing, planning of human settlements and environmental management (Eades, 1993; Soeters, 2012; Yakubu, 2021).

In the immediate post-independence period, state-building in Ghana sought to reduce the role of traditional authorities, in part due to their historic role in enabling colonial administration and power, and because they were seen as an obstacle to democratic and technocratic governmental control, whether under civilian or military rule. In the late 1960s, a political settlement began to emerge in Ghana in which traditional authorities came to occupy a more visible place in the governance of regions and cities (Tieleman & Uitermark, 2019). The 1969 and 1992 Ghanaian Constitutions gave recognition to the role of chieftaincy, later anchored in the Local Governance Act (2016) which recognised traditional authorities as key stakeholders in a decentralised local governance system.

Through this historical process, the power and roles of traditional authorities have evolved, with a distinction typically made between statutory and non-statutory roles (Różalska, 2021). Traditional authorities play a role in local government by being consulted in the nomination of the Metropolitan, Municipal and District Chief Executives, including the nomination of 30% of the membership of Assemblies.^2^ Even though the 1992 constitution barred chiefs from engaging in open and active partisan politics, they remain a formidable force for mobilisation of the population, a fact not lost on politicians (Owusu-Mensah, 2014). Less formally, traditional authorities are today primarily concerned with land management and as gatekeepers between formal institutions of the state and local people (Tieleman & Uitermark, 2019). Abdulai argues that chiefs have a variety of managerial roles, including advocacy on behalf of communities, dispute resolution, and resource allocation, including community responses to natural hazards, such as flood events (Abdulai, 2006). Boateng and Larbi show that chiefs have a role in the conservation of communal natural resources, such as use and harvesting of perennial trees (Boateng & Larbi, 2021).^3^

A third leg in the historical evolution of hybrid governance has been the role played by religious leaders in Tamale. Religious leadership was occasionally instrumental in the city’s governance, as exemplified by successful campaigns waged by Muslim clerics to stop demolition and forced evictions of residents of an Indigenous neighbourhood (Ward D) to pave the way for commercial use (Yakubu, 2021). Like the traditional arm, the religious arm remains active and relevant in Tamale. Religious leaders are widely considered to possess the moral authority to moderate city governance in Tamale. Local government authorities therefore find it expedient to engage religious leaders in decision-making and dispute resolution. Through consultation, religious authorities can play an important mediating role between competing interests at the local level. Over the past 40 years, local and international non-governmental organisations have also come to play complementary roles in governance in Tamale, including in the provision of expertise, financial resources and services.

Challenges that have come with the city’s growth are complex and entrenched. There is generally under-provision by the state of urban infrastructure and services, including roads, housing, health care, sanitation, reliable supplies of safe water and flood protection (Codjoe et al., 2020; Kayaga et al., 2021; Yakubu & Spocter, 2020). For example, the city’s population’s access to toilet facilities and improved sanitation is low compared to other Ghanaian cities (Fuseini & Kemp, 2016). Tamale has consistently ranked low in Ghana’s District League Table^4^ (DLT) in access to improved sanitation, even among the smaller towns in the Northern Region (National Development Planning Commission, 2023). This leaves a gap in provision, often filled by private and civil society action. For instance, residents, acting separately and in neighbourhood groups, provide and maintain domestic waste facilities. Chalfin describes similar resident-led initiatives in Tema (Ghana) for the provision, repair, and management of waste facilities (Chalfin, 2023). In Tamale, Sama Sama, a WASH-oriented NGO that partnered with the Metropolitan Assembly to deliver toilets to low-income households, with TaMA providing a subsidy. Sama Sama subsequently also developed water tanker services to deliver affordable and safe water to households.

In sum, public service provision in Tamale is polycentric, overlapping and dynamic. There are multiple sources of authority and resources at metropolitan, district and community levels, both complementary and in tension with each other. Within this governance constellation, traditional and religious authorities play a significant ordering role by governing key resources such as land, convening local interests, typically with reference to cultural practice and customary knowledge, and by mediating conflicts and disputes. The standing and legitimacy of traditional authorities are conditioned by their capacity to carry out these roles effectively, from the perspective of diverse local communities, which may have unequal access to traditional authority.

## 4 Methods

The data for this study were obtained as part of a broader research project *on Pathways to Equitable Healthy Cities* funded by Wellcome Trust (2018-24). The paper draws on long-standing historical and empirical research conducted by Fuseini and others on urban development of Tamale (Yakubu, 2021). In November 2022 we held a one-day workshop in Tamale with 50 local stakeholders to discuss issues related to a combination of research methods including personal and focus group interviews, multi-stakeholder consultations, field observations and published material on Tamale (see Annexe 1 for list of participants). The aim of the workshop was to create a dialogue with a range of local stakeholders (institutional and non-institutional actors) to identify key development and health issues for Tamale in the context of climate change, and to explore effective policies and interventions. Four main themes emerged: flooding and health; heat and health; food, nutrition and health; and access to safe drinking water, with a consistent view expressed by local stake-holders of a need to overcome an ‘implementation gap’ between existing policies and plans and observed action in communities.

As a prelude to the workshop and field research, the Project Team paid a visit to the Chief of Tamale (Gulkpe Naa) to inform him of the Team’s mission and to seek his guidance and permission. The Team discussed issues on climate change and health and obtained insights regarding traditional knowledge on climate change and flooding with the Gulkpe Naa’s council of elders. In April and May 2023 further fieldwork was carried out in Tamale involving 15 interviews with expert stakeholders (people working in flood management), and 6 focus groups with chiefs and community opinion leaders. It draws on expertise of the author group in flood risk. Field observations were also undertaken (see Figure 1). The research and management in diverse contexts, in particular employing prevailing engineering concepts and practices. Also draws on published sources on long-standing historical and empirical research on urban development in Tamale (Fuseini, 2021; Fuseini & Kemp, 2016).

## 5 Flood risk and resilience governance

### 5.1 National context

National, regional, and metropolitan institutions make up the three tiers of Ghana’s disaster risk management system. National government sets development frameworks, policies, and national programmes. Regional governments are responsible for coordination between municipalities, while Metropolitan and District Assemblies are responsible for local planning and implementation.^5^ TaMA has a range of agencies contributing to flood governance in Tamale: the Hydrological Authority, the Meteorological authority, the Physical Planning Unit, Land Commissions, Urban Roads, Waste Management Department, Works Department. National agencies, principally the National Disaster Management Organisation (NADMO), Environmental Protection Agency and Hydrological Services Authority support implementation at the local level (Amoako & Inkoom, 2018). Despite a density of institutional roles, flood protection and longer-term adaptation have been given limited attention in city plans. A review of the Tamale Metropolitan Medium-Term Development Plan revealed limited inclusion of flood and flood adaptation strategies. Planning has tended to be ad-hoc and reactive (Aboagye et al., 2020; Kayaga et al., 2021).

### 5.2 Role of traditional and religious authorities

In the context of polycentric governance, traditional authorities fulfil diverse functions in the co-production of public services, notably in flood resilience and protection. Their roles encompass the provision of knowledge, physical and cultural resources, project evaluation, and conflict resolution. However, the legitimacy and influence of traditional authorities are contingent upon their interactions with other stakeholders, particularly local and national governmental bodies. This intricate network of actors introduces both complexity and opportunities within the city’s governance framework. Citizens engage in a strategic process known as ‘institutional shopping,’ whereby they navigate between different components of the governance structure, seeking solutions that align with their interests and needs (Nchanji & Bellwood-Howard, 2018).

When it comes to flood risk management and adaptation, chiefs and traditional leaders are expected to leverage their influence and cultural knowledge to spearhead initiatives that safeguard communities from flooding. One of the primary expectations placed upon chiefs and traditional leaders is their role in community engagement and education. They are looked upon to mobilise community members, impart knowledge about flood risks, and galvanise collective action towards preparedness and adaptation measures. But they do this without specific financial resource or legal authority. Moreover, while traditional authorities possess cultural knowledge about local environmental conditions and traditional coping mechanisms, their lack of technical expertise in modern flood risk assessment and management techniques can be a significant barrier. This can hinder their ability to assess flood risks, help prioritise interventions, and implement appropriate adaptation measures tailored to the unique challenges faced by Tamale’s rapidly evolving urban landscape.

Institutional constraints further complicate the picture. While chiefs wield significant influence within their communities, bureaucratic barriers and jurisdictional conflicts hinder collaboration with formal government institutions responsible for flood management. This lack of coordination can lead to disjointed efforts and inefficiencies in resource allocation, ultimately diminishing the impact of flood risk management initiatives. Social and cultural norms within Tamale’s communities also pose challenges to inclusive decision-making processes. Hierarchical structures and gender biases can limit the participation of marginalised groups in flood management efforts, leading to solutions that overlook the needs of certain vulnerable populations. Chiefs may find it challenging to adapt quickly enough to these evolving environmental conditions and may struggle to influence land use decisions and development regulations effectively.

Collaborative approaches that bridge the gap between traditional and modern knowledge systems, capacity-building initiatives that empower traditional leaders with the necessary technical skills, and inclusive decision-making processes that prioritise community interests would help strengthen multi-level community resilience. By overcoming these obstacles, chiefs and traditional authorities foster flood-resilient communities.

#### 5.2.1 Mediating formal knowledge and traditional practices

In Ghana, significant scientific and engineering research has been conducted on flooding, particularly in coastal cities like Accra, Cape Coast, and Sekondi-Takoradi. In addition, national assessments and more detailed studies on the dynamics of flooding have been conducted (Almoradie et al., 2020; Chrysogonus et al., 2022). Despite the existence of these assessments, their impact on flood protection and resilience measures has been limited. Flood management interventions have typically been reactive rather than proactive. For instance, in response to severe flooding in 2017 that resulted in fatalities, a large storm drain was constructed along the Bolgatanga Road in Tamale.

While there is high-level technical expertise on flooding in Ghana, in both the public and private sectors, effective flood management strategies have been piecemeal, with interventions often prompted by crisis rather than informed proactive measures. The first comprehensive city-wide flood risk assessment was carried out for Tamale in 2023, utilising the HEC-RAS model, with funding provided by the UK Foreign, Commonwealth & Development Office (Sharma et al., 2023). Typically, external technical consultants support flood risk mapping efforts, employing hydraulic and/or hydrological models to predict flood dynamics under various rainfall scenarios. These assessments incorporate population data, including vulnerability indicators, to gauge potential impacts (Bogardi et al., 2021).

However, creating and validating these models requires extensive data, related to topography, rainfall patterns, water levels and velocities in channels, floodwater depths, and socioeconomic and demographic distributions. Unfortunately, for Tamale, much of this crucial data are lacking, especially at high resolutions. Notably, the city lacks rainfall and water level monitoring systems, and satellite imagery cannot accurately measure the extent or depth of short-duration flash floods commonly experienced in the area. In the absence of comprehensive data, flood protection and resilience efforts in Tamale rely heavily on local knowledge and observations. Within local communities, people frequently monitor water levels in channels and observe flood events, using environmental cues such as high-water marks on buildings to estimate the depth and extent of flooding. Farmers possess valuable insights into rainfall patterns before and during floods, drawing on their experiences to adapt planting and fertilisation strategies. Chiefs and elder groups often have detailed knowledge about vulnerable individuals and communities, aiding assistance efforts (Membele et al., 2022).

Communities contribute to flood resilience efforts by sharing knowledge about historical flood events and the evolution of their settlements (Ziga-Abrotta et al., 2021). This collective knowledge, combined with modern scientific approaches, could aid in the development and validation of flood risk models, especially in areas where data limitations pose significant challenges (Echendu, 2023). Indigenous adaptive measures embraced by residents in Tamale reflect a blend of traditional wisdom and contemporary techniques geared towards lessening the impact of floods. These measures underscore the resilience and ingenuity of Tamale’s communities in confronting recurrent flooding episodes. By integrating age-old practices with modern approaches, residents endeavour to shield their homes and means of sustenance from the detrimental consequences of floods (see Table 1). In doing so, they bolster their ability to adjust to shifting environmental realities and fortify their resilience against future challenges.

#### 5.2.2 Governing land and natural resources

Spatial planning and land management are critical to flood risk and flood protection. Where activities are located will determine exposure of households and business to flood risk, flood protection measures require space and lead to water flows and storage which also require space. Finally, more systemic adaptation to flood risks – for instance rezoning and resettlement – requires the reconfiguration of urban space. Land in Dagbon and Tamale is customarily held in stewardship by chiefs. New development and change of use would typically require agreement from traditional authorities. Chiefs and elders may also play an advisory role, bringing to bear customary and practical knowledge relevant to rights and use of a plot of land. This makes chiefs important players in planning and land governance in Tamale (Akaateba et al., 2021; Fuseini, 2021). The role of the formal planning function of TaMA appears to be compromised. In principle, land use management is handled through a dual system, with plans first being prepared by the Metropolitan Planning Department, reviewed by traditional authorities before being passed on to the Metropolitan Planning Authority, through the Metropolitan Planning Co-ordinating Unit, for approval. This last step is often absent. In 2022 just 40 applications for development permits were processed by the Tamale Planning Department,^6^ suggesting that most development is not covered by statutory planning guidance and controls. This does not imply that statutory control is ignored, since chiefs may be acting to reconcile customary practice with statutory guidance, acting as intermediaries and enablers of government policies.

Chiefs’ control over land management makes land relatively accessible to diverse social and economic strata in the city, but it may also be exclusive. Customary land management cuts across conventional markets for land and, in principle, non-financial values can play a significant role in land-use decisions. In practice, chiefs extract tribute and other payments through land transactions. Mostly they are motivated to extract the highest rents, even at the risk of alienating familial, religious and ethnic groups whose support they depend on. In some areas, chiefs act to reserve land for open spaces, green areas, buffer zones and other public works in Tamale (Fuseini & Kemp, 2016) and limit access to flood-prone areas for development using historical and customary knowledge. This does not mean that such areas remain undeveloped through encroachment by informal settlements, with weak enforcement of zoning by both planners and traditional authorities. Chiefs have a more clear role in disaster response. For example, following the devastating flood in the city in 1989, when an old dam near the city waterworks broke its banks, chiefs worked with the NADMO and local communities to secure land to resettle affected families (Fuseini & Kemp, 2016). This resettlement stimulated substantial urban expansion to the north-west of the city. Some residents later rebuilt dwellings in flood-prone locations closer to the city centre as second homes.

#### 5.2.3 Project appraisal and dispute resolution

Rules and procedure are central to public sector resource allocation and to the appraisal of private development proposals. Typically, this includes a comparison with statutory planning guidance, a benefit-cost or risk assessment and a process for review that is open to interested and affected parties. In Ghana, these rules and procedures are codified in law (Act 936 of 2016), creating opportunity for substantive or procedural challenges by organisations and citizens, and possible adjustments. In polycentric, hybrid governance systems, dual project appraisal and review processes operate – one governed by traditional authorities, the other by statutory authorities – with a tighter or looser coordination between these parallel and complementary systems. Residents in under-provisioned neighbourhoods have demanded infrastructure and services from TaMA directly. Such provision, of roads, power and flood protection, has generated local struggles because settlement upgrading may be associated with demolition and evictions (Yakubu, 2021). Traditional authorities are vital in acting as an intermediary in the design of infrastructure schemes and in dispute resolution in neighbourhoods.

The dual system of authority in Tamale introduces complexity and ambiguity since overlapping rationalities and governance approaches create the need for additional negotiation and dispute resolution. The greater opportunity for claimants to appeal to alternative sources of authority (‘institutional shopping’) acts to open-up processes to a wider range of interests and may improve the design and acceptance of development projects. But it can also act as an impediment to decision-making. A widely held assessment among flood risk stakeholders in Tamale is that an ‘implementation gap’ exists, partly due to lack of financial resources and institutional capacity, and also due to the complexity of existing hybrid governance and the new opportunities for sectional contests which this introduces.

## 6 Towards integrated flood management and resilience in Tamale

Our analysis highlights three features of flood risk management in Tamale. First, the combined effects of the city’s growth and climate change are rapidly changing patterns of flood risk and vulnerability. Second, the prevailing governance regime in Tamale through which these risks are understood and responses are developed involves a complex interaction between state and traditional authorities. Third, there is a widely held perception in Tamale communities that an implementation gap exists between the action required to reduce flood vulnerability and build resilience and what is observed on the ground. How can more effective and equitable flood management and resilience be organised? Our starting point is the social and political context of hybrid governance as it conditions intersecting knowledges, access to resources, project appraisal and dispute resolution. We have also seen that a nested series of responses will exist in any place, starting with the private action by households, through collaborative responses by communities – typically enabled by local NGOs who may be enabled by international donors – and complemented by broader-scale responses, including early warning systems, engineered structures, nature-based solutions and non-structural measures in which the state plays a more central role, through the provision of expertise, finance and planning control. We turn now to these broader responses and how they may be designed and implemented in the context of hybrid governance in Tamale.

### 6.1 Engineered structures

Hard engineering approaches to flood management involve constructing artificial structures to manage where, when, and how flood waters move. These approaches have been central to technocratic flood management strategies, including in Tamale. Whilst it is increasingly recognised that hard engineering approaches alone are inadequate to manage flooding under climate change, they continue to be an important tool. Storm drains are the most common example of hard engineering approaches in Tamale which, since the construction of the first drains in the 1970s, have evolved into an extensive network throughout the city (FCDO, 2023).

**Figure 2:**
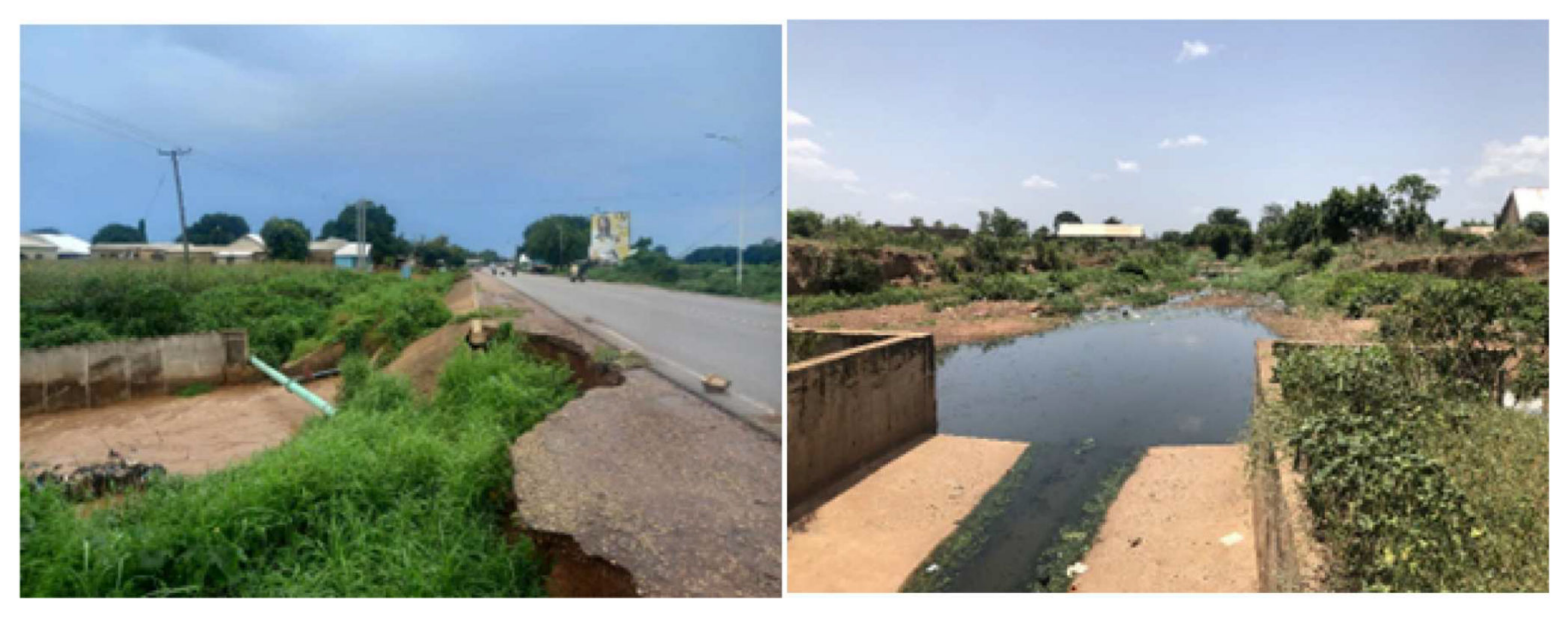
Physical condition of engineered storm drains in Tamale (2023) (in supplemental material).

By moving water around, storm drains displace flood exposure from one place to another. This strategy is effective at reducing risk when exposure is displaced from an area of high value or vulnerability, such as a densely populated and economically active city centre, to one of lower value and vulnerability, such as sparsely populated agricultural land. However, when poorly designed or constructed, or subject to conditions for which they are not designed, storm drains can exacerbate flood risk on a city level. For example, when the capacity of the drainage network is not sufficient to cope with the volume of water generated during a rainfall event, water will overtop the banks, or get backed up at culverts and bridges, causing localised flooding along the watercourse, amplifying risk for more vulnerable places and people. This has emerged as a problem in Tamale. For example, some tributary drains are lower than main channels, directing flood water back towards communities, and others are poorly constructed, with several instances of collapse around the city (Figure 2).

The national Hydrological Authority and Department of Urban Roads manage design, construction, and maintenance of storm drains. Their connection to centralised specialist authorities allows them to leverage technical expertise and the significant resources required. However, design and construction are typically contracted with third parties. Whilst cost-effective in the short-term, this setup leads to disconnected programmes and poor-quality work, enabled by weak auditing and quality control by TaMA. This is further exacerbated by delays or defaults in paying contractors, leading to unfinished works and challenges in filling contracts.

Although traditional authorities often lobby government to build more storm drains to protect their communities, they are rarely consulted on their design or involved in their construction or maintenance. They could make significant contributions and help overcome current failures. Traditional authorities could contribute to the design of the drainage network on a city and local scale. They could be consulted about decisions on the spatial distribution of drains and contribute to analysis of failures in the current network. On a local level, they could help identify natural channels that may appear only during flood, and may be suitable for engineered drains. Planning and design of works by traditional authorities with state authorities could help raise the levels of trust for contractors, ensuring that contracts are fulfilled, although this must be backed up by reliable funding from national government. Finally, traditional authorities could contribute to quality control of construction work and identify maintenance requirements.

### 6.2 Nature-based solutions

Nature-based solutions to flood management involve protecting, managing, and restoring natural process to affect how flood waters are generated and where and how they move. Whilst these strategies have been used in water resource management for centuries, only in the last two decades have they been scientifically tested and applied in diverse contexts (Ochoa-Tocachi et al., 2019). Their aim is usually to reduce the volume of surface run off generated during rain events, to slow water down along its path to reduce peak flows, and to stabilise and protect ecosystems like riverbanks (Hartmann et al., 2019; Acreman et al., 2021). Common examples include afforestation to reduce surface run off, and temporary water retention to distribute storm water throughout the network more slowly.

In Tamale, large volumes of surface water can be generated rapidly during rainfall events, owing to large areas of impervious surfaces and increasing frequency and intensity of rainfall. Storm drains and rivers are often overwhelmed by these flash floods, causing localised flooding. Addressing these challenges by building higher capacity storm drains alone is prohibited by expense and practicalities such as space and topography. An alternative approach could be to reduce the generation of surface runoff in the first place. This could be achieved by increasing the proportion of land area covered by trees and vegetation, which can serve to increase the infiltration capacity of soils and surfaces, and intercept rain before it reaches the ground (canopy interception) (Acreman et al., 2021). These measures can also slow down transport of surface waters to channels, mitigating peak flows in drains. There may also be significant co-benefits, such as protection from extreme temperatures, increases to biodiversity, and the sustainable production of timber and fuel.

These measures are well-understood and advocated by planners in Tamale. Increasing the tree cover could be realised by either concentrated (e.g. whole wooded areas like forests) or distributed (e.g. urban trees or gardens) efforts, or a combination of the two. The diversity of approaches to increase tree cover presents opportunities for contributions by several actors (Kuller et al., 2022). Planting or preserving whole forested areas requires significant land area, usually provided by traditional authorities, although some private- and state-owned land may be suitable. For example, approximately 100 ha of undeveloped land close to the city centre (the Salaga Road and St Charles Road) is owned by Tamale Teaching Hospital. Additionally, property owners and communities could contribute by planting and protecting trees. To mitigate the high cost of saplings, local government could facilitate a link between a city-wide tree planting programme developed in concert with traditional authorities, with the national ‘Green Ghana’ programme, which aims to plant 5 million trees with funding from the United National Capital Development Fund.

Protecting trees remains a primary challenge for nature-based approaches in Tamale. Competition for land and fuel makes them vulnerable to degradation and extractive practice. For example, in the Nobisco forest in the north of the city, encroachment by roadside properties is evident. The Forestry Commission is led by competent and determined officials who protect much of the city’s forests through patrols and prosecutions,^7^ although their protection extends exclusively to the forests they manage. Traditional authorities already play a customary role in protecting trees. According to traditional practices, whilst land can be allocated to individuals, companies, and the state, the trees always belong to the chiefs (‘you own the land, but you don’t own the trees’).^8^

Effective and adaptive forest management is needed to sustain and extend nature-based solutions. The Forestry Commission hosts significant forest management expertise, as well as the equipment and people necessary to carry out most works, such as thinning and restocking. However, officials typically adopt Western forestry theory and practice, and may employ practices that are sub-optimal for local contexts (e.g. planting primarily non-native trees) (Acreman et al., 2021). Traditional authorities, on the other hand, may possess significant indigenous and local knowledge about suitable trees and forest management. A combination of these approaches could maximise the efficacy of forest management in Tamale, improving the provision of nature-based solutions and other benefits like biodiversity (Sraku-Lartey, 2014).

### 6.3 Information and behavioural measures

While the focus in conventional flood management is often on making structural changes to the environment to control flood waters, non-structural measures can be vital in mitigating the severity and impacts of flooding. These include behavioural changes that focus on the actions of people in preparing for, responding to, and recovering from flood events. Their aims are varied, ranging from reducing flood drivers in given areas, reducing the exposure of people and property, and mitigating negative impacts. Common examples include early warning systems, which can allow people time to prepare or evacuate, and improved waste management. The latter is recognised as fundamental to effective flood management in Tamale in several recommendations and plans. Solid waste, especially plastics, is often discarded directly in drains, or disposed of indiscriminately where it is transported by surface waters into drains. Blocked drains cause backed-up storm waters and localised flooding. Interviews and focus groups with state and non-state authorities confirm that both are acutely aware of the link between waste and flood risk and motivated to affect change. Both authorities have programmes addressing this challenge – the TaMA Waste Management Department and a local chief who leads policy and planning of waste management in the city – although these appear to work in parallel rather than together.

Several conditions are required for effective waste management. Designated collection sites are needed for households and business to leave waste (bins or local tips) that are affordably accessible. Waste must be routinely collected and transported to management and disposal facilities. Organised household waste management requires substantial resources and capabilities and transport infrastructure. It requires land for facilities and low risk from natural hazards like floods and fires. Delivering this complex managed system often representing the highest expense for local administrations in low- and middle- income countries, typically comprising up to 20% of municipal budgets (Kaza et al., 2018). A vital condition of effectiveness is that citizens engage with the system. Traditional authorities could play several roles in co-producing effective municipal waste management in Tamale. They could facilitate community-level involvement in the design and implementation of collective waste management, including appropriate behavioural measures, framing and reinforcing messages in their communities. Chiefs could play a regulatory role in endorsing ‘good’ waste behaviours by households and businesses, and they could make available land for waste facilities.

### 6.4 Summary

Table 2 provides a summary of the discussion above. We find that engineered, nature-based and information instruments for flood resilience are not equally well-known and trusted by planners and communities in Tamale. We also find that there are significant opportunities for traditional authorities to play a role in designing, implementing and governing flood resilience, including the full range of approaches potentially available – engineering, nature-based and behavioural. A well-constituted set of hybrid governance arrangements therefore offers the promise of both a broader set of approaches to be adopted in Tamale, and for these to be more effective, legitimate and fair. A ‘double dividend’ could therefore be generated from better established and resourced hybrid governance arrangements, adoption of a wider set of tools for flood resilience and greater engagement and acceptance of these measures by vulnerable communities.

## 7 Conclusions

The interaction of state and traditional authorities leads to the ‘co-production’ of flood risk and resilience in Tamale (Ostrom, 1996). But there are complexities and ambiguities in the articulations of these two very different governance systems’ leading to fragmented and unequal outcomes for communities who, while disempowered from public decision-making, are often forced to make private provision to build *ad hoc* resilience to flood damage and risk. The governance challenge is to equip and enable the dual authority of state and customary power to produce adaptive outcomes in the context of urban growth and climate change. Our approach in this paper contrasts with the more conventional approaches which emphasise the strengthening of state institutional capacity to deliver technocratic solutions. We argue that this does not reflect the social realities in the city, has been ineffective in the past and fails to draw on local knowledge and resources. Traditional authorities play an ambiguous, under-recognised and largely unsupported role in flood protection through the provision of customary and practical knowledge, key resources (through the control of land management) and through local dispute resolution processes.

How can traditional authorities play a more effective role in co-producing flood resilience? One route would be to enable technical capabilities among traditional authorities. This is already happening through the growth of a tradition of ‘return chiefs’ – highly-educated chiefs with international experience able to leverage broader networks for expertise and money (Kleist, 2011). We also observed this in our meeting with elders at the Gulkpe Naa’s palace, where the conversation was led by the Chief’s Secretary, an administrator at the Tamale Technical University (TaTU) acting as an intermediary between traditional and technical knowledges. A second approach would be to create clarity and build incentives for chiefs to act as intermediaries and moderators of government policies. Currently, the semi-formal and partially-defined role Chiefs in flood governance is primarily through consultation on applications for development and changes in land use. In practice, dual authority over land management is mostly inactive, with traditional authorities, as customary owners of land, having primary decision-making power over most land-use changes. This has the effect of undermining planning by metropolitan authority who remain accountable for outcomes. A third approach would be to give a formal role, possibly with public funds, to traditional authorities in assessing flood risk, drawing on community knowledge, and in the design and maintenance of flood risk and resilience planning. New structures and processes may be needed that bring together traditional and technical knowledge and authority. Community durbars have long been used in Ghana to promote public health and can play a role here. The aim would be to enable the adoption of the full range of engineering, nature-based and behavioural responses available. Chiefs already play an active role in planning by shaping and contesting the content of metropolitan plans. The governance challenge is to design consultation and decision-making processes that openly and effectively intermediate between traditional and state authorities without seeking to mix or integrate them. They remain separate worlds, but they need to be put in dialogue with each other.

Any dual system of governance risks imposing new coordination and political costs to the governance and delivery of collective goods and services. But given the weaknesses of current state-based provision of services and the social reality that choices, behaviours and outcomes are shaped by an often-ambiguous hybrid governance, there is a need to look for more transformational alternatives, especially in the face of mounting flood risks because of climate change. Conceiving of flood risk management and resilience as being co-produced by public and traditional authorities, working to reduce community vulnerability and build community resilience, provides a renewed basis for a more adaptive, fair, and legitimate governance of flood risks, now and into the future.

## Supplementary Material

Supplemental Material

## Figures and Tables

**Table 1:** Household and neighbourhood flood risk adaptation measures adopted in Tamale (authors’ field research).

Flood adaptation	Description
*Re-roofing Houses*	Before the rainy season begins, residents ensure that their roofs are in good condition by re-roofing them. This proactive measure helps limit water seepage into homes, reducing the risk of damage to property and belongings.
*Replastering Mud Houses*	Residents living in mud houses (locally known as ‘sugar water houses’) take steps to reinforce their structures by replastering walls. This helps ensure that the houses can withstand intense rainfall. An increasing number of individuals are opting to use cement for plastering, strengthening structures and providing better protection against water infiltration.
*Sandbags*	Sandbags are used to redirect water flow away from homes and properties. Residents can create barriers minimising flood damage.
*Building Barriers*	Some residents construct barriers and utilise piers to mitigate the impact of floodwaters. These barriers can redirect water flow or provide additional support to vulnerable areas, such as entrances or low-lying sections of properties.
*Raising Foundations*	Foundations of homes can be raised from the beginning of the construction process. Elevating foundations reduces the risk of structural damage.
*Elevating Electrical Appliances and Furniture*	Residents routinely elevate electrical appliances and furniture above potential flood levels, safeguarding these from water damage.
*Improving Drainage Systems*	Regular maintenance of drainage channels and gutters is a common practice, minimising the risk of flooding. Some residents have installed additional drainage infrastructure to divert excess water away from their properties.
*Information Campaigns on Flood Preparedness*	Ongoing educational campaigns on flood preparedness are conducted by NADMO, building awareness and skills to respond effectively to flooding emergencies. Training programmes cover evacuation procedures, first aid, and effective communication strategies.

**Table 2:** Overview of collective approaches to flood management and their associated actors, conditions, barriers, and opportunities in Tamale, Ghana.

Approach	Engineered	Nature-based	Information and behavioural
**Description**	Structural measures typically requiring heavy engineering (e.g. concrete) to divert or protect against flood waters.	Structural measures typically involving low impact, ‘green’ interventions that aim to reduce surface run off and slow water down along its path.	Behavioural changes that focus on the actions of people in preparing for, responding to, or recovering from floods.
**Examples**	Storm drains; Road gutters; Levees; Dams.	Tree planting; Riverbank protection; Sustainable drainage systems; Flood retention areas.	Early warning systems; Evacuation planning; Risk mapping; Re-zoning; Waste management.
**Actors**	Substantial investments in Tamale. Primarily resourced and designed by state agencies, usually with limited consultation of traditional authorities.	Not common in Tamale. Typically requires a hybrid approach with state and non-state actors collaborating closely.	Fragmented implementation in Tamale. Often requires a hybrid approach with state and non-state actors collaborating closely.
**Conditions**	Requires significant financial resources, technical design by experts, planning and maintenance by local state.	Low cost and drawing on traditional knowledges, requires redesignation of land-use, consultation with communities and hybrid governance at local level. Requires protection from encroachment by competing land-uses.	Complex enabling systems need to be in place (e.g. early warning systems, waste collection), mainly through state agencies. Behavioural responses depend on community engagement and trust. Enforceability locally-mediated.
**Challenges**	State funding often piecemeal, poorly implemented projects causing spill-over risks for poor people. Requires active maintenance and may have a short longevity. Often not resilient to climate change.	Largely unknown and likely not understood and trusted by many citizens. Liable to damage (e.g. deforestation), and/or land-use change.	Long-term investment and governance required for effective systems. Substantial communication and engagement with households and communities needs to be regular and continuous.
**Opportunities for engaging traditional authorities**	Favoured by many in Tamale. Chiefs and communities have detailed local knowledge that could help design and appraisal of schemes. Could enable community engagement in design, appraisal, and maintenance as element of more integrated flood resilience.	Transcends scale limitations – potentially workable at household to landscape scales. Cumulative effects so can start small and scale up as acceptance, land and resources become available. Traditional authority over land-use decisions and communally-held resources (e.g. trees) gives them an important role in enabling NBS.	Information-behavioural interventions require coordination, understanding and trust. These can be provided in communities by traditional authorities. Also, potential for customary knowledge to play a role in early warning system design and implementation. Chiefs and religious leaders could play a role in framing and enforcing new behaviours (e.g. through Durbars).

## Footnotes

1
**Notes**
Its name is derived from the word *Tamani*. In Dagbani language, ‘tama’ means the shea fruit.

2 The structure of traditional authority in Tamale is through a hierarchy of chieftaincies. The city is situated in the Kingdom of Dagbon whose overlord is the Yaa Naa Abubakari Mahama II. He has decreed that his representative in Tamale is the Naa Yaba Gulkpe Naa, the Paramount Chief of the Gulkpegu Traditional Area, although this is also disputed (https://dailyguidenetwork.com/ya-na-clarifies-status-of-gulkpe-na-dakpema/).

3 For instance, community and village-level chiefs (Dohin-Naa and Tuu-Naa) harvest African Locust Bean (*Parkia biglobosa*, dawadawa) trees used for nutrition and medicinal value in their areas of jurisdiction. This is strictly enforced and conflicts may arise if people do not respect this arrangement.

4 An annual assessment of the impact of service delivery across all 261 Metropolitan, Municipal and District Assemblies. The assessment covers seven sectors including health, nutrition, education, water, sanitation, governance, and energy. About 22 indicators are used to calculate an index which determines the rank of a District.

5 Regional and local Governments in Ghana have restricted revenue-raising powers, so all major funding for Government agencies come from national tax and non-tax revenues.

6 Chief Planner, TaMA, Pathways workshop, Tamale, 10th November 2022.

7 With fines for those who fell trees without consent.

8 Traditional practice holds that land cannot or should not be sold but remains a common good.
