# Supplementary material for: Chiefs and floods: hybrid governance and co-production of flood risk adaptation in Tamale, Ghana1
Source: J Environ Policy Plan. Author manuscript; Available in PMC 2024 Nov 28. (PMC11552701; doi:10.1080/1523908X.2024.2410899)
Supplement: Supplemental Material [file EMS199882-supplement-Supplemental_Material.docx]

SUPPLEMENTAL MATERIAL

FIGURE 1


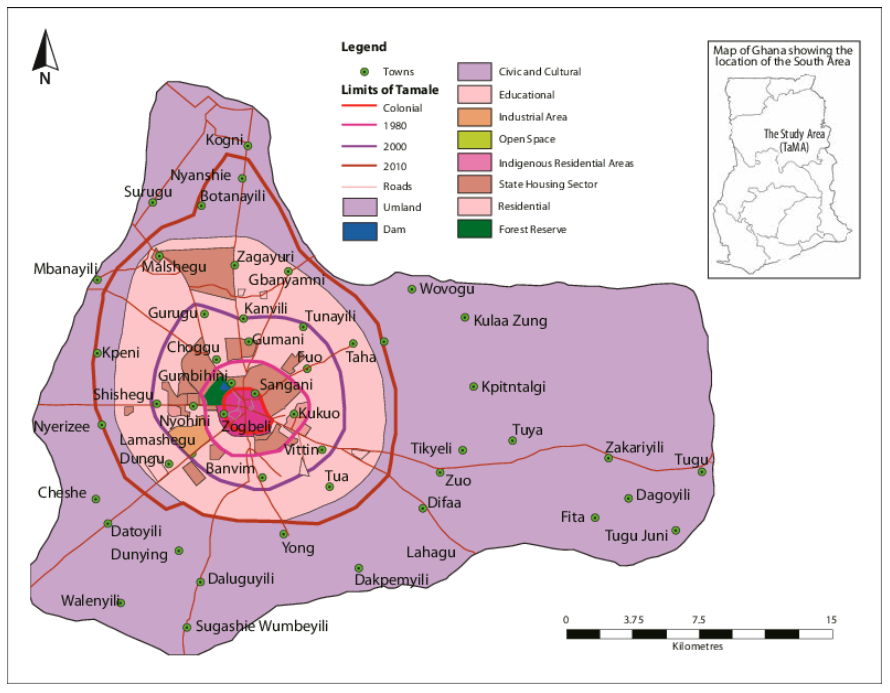


Figure 1: Map of Tamale

FIGURE 2


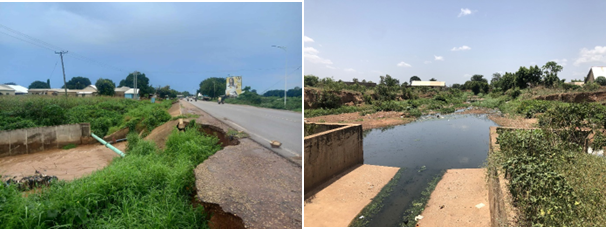


*Figure 2. Left: Poor construction of the drainage network, in this case the failure to backfill with suitable material, has led to collapse, posing significant risks to the public. Right: An unfinished or poorly designed drainage network has led to severe flooding for some communities.*

**Annex 1: PATHWAYS TO HEALTHY EQUITABLE CITIES project, Workshop on Climate Change, Health and Flooding,**

8-11 November 2022,

**Participants list**

| **Type of Actor** | **Name of Organisation** | **Designation** |
| --- | --- | --- |
| Government | Tamale Metropolitan Assembly | 1. Regional Coordinating Director |
|  | Physical Planning Department | 1. The Tamale Metropolitan Physical Planning Officer |
|  | Hydrological Services Department | 1. Regional Hydrologist, Tamale |
|  | Department of Urban Roads | 1. Regional Engineer, Tamale |
|  | Land Commission | 1. The Regional Land Commissioner, Tamale |
|  | Forestry Commission | 1. Chief Executive Officer, Tamale |
|  | Community Water and Sanitation Agency | 1. Deputy Regional Director, Tamale |
|  | Ghana Meteorological Agency | 1. Regional Director, Tamale |
|  | National Disaster Management Organisation (NADMO) | 1. South-Sub metro officer, Tamale 2. South-Sub metro Administrator |
|  | Northern Development Authority | 1. Northern Regional Director, Tamale |
|  | Council for Scientific and Industrial Research - Savanna Agricultural Research Institute (CSIR-SARI) | 1. Principal Researcher |
| Non-Governmental | Send Foundation | 1. Project Manager, Salaga |
|  | Yaro (Youth Advocacy on Rights and Opportunities) | 1. Chief Executive Officer, Tamale |
|  | SWIDA- GH  Savannah Women Integrated Development Agency | 1. Executive Director, Tamale |
|  | Tree Aid West Africa | 1. Chief Executive Officer, Tamale |
| Private | Amazing Farms | 1. Farmer/Teacher |
| Academia | University for Development Studies | 1. Professor, Department of Applied Economics 2. Professor, Department of Economics 3. Lecturer, Department of Environmental and Occupational Health |
|  | Tamale Technical University | 1. Lecturer, Department of Natural Resources, Tamale 2. Lecturer, Department of Hospitality and Tourism Department |
|  | University of Ghana | 1. Professor, Department of Geography and Resource Development 2. Professor, Centre for Migration Studies 3. Professor, Department of Earth Sciences 4. Lecturer, Department of Geography and Resource Development 5. Senior Lecturer, Department of Adult Education &Human Resource Studies. 6. PhD Candidate, Department of Geography and Resources Development. 7. PhD Candidate, Department of Geography and Resources Development |
|  | King’s College London | 1. Professor, Department of Geography |
|  | Harvard University | 1. Associate Professor, T.H. Chan School of Public Health |
|  | Imperial College, London | 1. Professor, Department of Civil and Environmental Engineering 2. Research Associate, Department of Civil and Environmental Engineering 3. Professor, Department of Civil and Environmental Engineering |
